# AMPylation: key roles in biological regulation across prokaryotes and eukaryotes

**DOI:** 10.3389/fcimb.2026.1763599

**Published:** 2026-01-29

**Authors:** Xinyi Wang, Junyong Yang, Zhaotai Zang, Yanan Wang, Zihan Shao, Bingqing Li, Haihong Jia

**Affiliations:** 1 Department of Pathogen Biology, School of Basic Medicine, Shandong First Medical University & Shandong Academy of Medical Sciences, Jinan, China; 2 Department of Clinical Laboratory, Shandong Provincial Hospital Affiliated to Shandong First Medical University, Jinan, China; 3 Key Lab for Biotech-Drugs of National Health Commission, Jinan, China; 4 Key Lab for Rare & Uncommon Diseases of Shandong Province, Jinan, China

**Keywords:** AMPylation, application, biological function, prokaryotes and eukaryotes, regulatory mechanisms

## Abstract

AMPylation, as a crucial post-translational modification, is widely present in both prokaryotes and eukaryotes, playing a key role in regulating biological functions. The regulation of biological functions by AMPylation is a complex and diverse process. In prokaryotes, AMPylation plays a role in processes such as self-metabolic regulation, gene expression control, and maintenance of cellular redox homeostasis. Eukaryotes utilize AMPylation to regulate endoplasmic reticulum stress, participate in disease progression, and modulate immune responses. During interactions between prokaryotes and eukaryotes, bacteria can influence host cytoskeletal function, anti-apoptotic processes, and vesicular transport through AMPylation, thereby enhancing their survival within the host. Currently, AMPylation has been applied in numerous directions, such as detecting modifications, constructing disease models, and studying protein functions. This article highlights the diverse roles of AMPylation in regulating biological functions and reviewed the application progress in various fields, aiming to provide theoretical foundations for understanding their mechanisms in pathogen control and eukaryotic disease prevention.

## 1 Introduction

Post-translational modifications (PTMs) involve the covalent attachment of one or more chemical groups or other proteins to specific residues on a protein. These modifications are extensively involved in various normal biological activities within the body and play an indispensable role in regulating bodily functions (Walsh et al., 2005). To date, over 400 types of post-translational modifications of proteins have been identified. These modifications play a crucial role in regulating gene expression in living organisms and have emerged as a highly significant field in international protein research (Khoury et al., 2011; Aebersold et al., 2018). Among these, AMPylation (adenosine monophosphorylation) modification, as a newly discovered PTM pathway, has garnered significant attention.

AMPylation can be traced back to 1967, when researchers in the Stadtman laboratory first reported that an unknown protein in *Escherichia coli (E. coli)* transfers AMP to glutamine synthetase in response to nitrogen concentration changes, leading to alterations in the enzyme’s catalytic activity (Shapiro et al., 1967; Brown et al., 1971). Subsequent studies further demonstrated that this modification is widely present in both prokaryotes and eukaryotes and is closely associated with numerous physiological and pathological processes. For example, the prokaryotic bacterium *Vibrio parahaemolyticus* promotes infection by inhibiting the normal function of the host cell cytoskeleton through AMPylation (Yarbrough et al., 2009).

The AMPylation modification process is a complex and orderly sequence involving steps such as protein recognition, catalytic reaction, and AMP group attachment. First, the AMPylator specifically recognizes the substrate protein through its unique structural domain. This recognition mechanism ensures modification precision, enabling the AMPylator to locate specific target sites among numerous proteins. Subsequently, in the presence of ATP, the AMPylator catalyzes the reaction of ATP molecules, forming a phosphodiester bond between the AMP group and the hydroxyl group of a specific amino acid residue on the substrate protein. This completes the attachment of the AMP group, resulting in AMPylation modification of the substrate protein (Yarbrough and Orth, 2009). This modification process is reversible. When the intracellular environment changes or protein function needs adjustment, de-AMPylase can catalyze the reverse reaction, removing the AMP group from the protein. This restores the protein to its unmodified state, enabling dynamic regulation of protein function (Ham and Orth, 2011; Tan and Luo, 2011; Preissler et al., 2017a, 2017b).

In the field of prokaryotes, research has revealed that glutamine synthetase (GS) activity can be regulated by AMPylation, thereby influencing nitrogen metabolism (Anderson and Stadtman, 1970; Brown et al., 1971). Furthermore, AMPylation participates in gene expression regulation and maintains cellular redox homeostasis in prokaryotes. These findings significantly expand our understanding of AMPylation’s functions in prokaryotic organisms (Harms et al., 2015; Stanger et al., 2016; Sreelatha et al., 2018; Wang et al., 2022). In eukaryotes, AMPylation often serves as a fundamental mechanism for maintaining cellular homeostasis, and its dysregulation frequently leads to various diseases. A growing body of research indicates that abnormal AMPylation is closely associated with the onset and progression of multiple diseases, such as neurodegenerative disorders, cardiovascular diseases, and cancer. This has positioned AMPylation as a hotspot in disease research (Fu et al., 2007; Sanyal et al., 2019; Chien et al., 2021; Rebelo et al., 2022; Perera et al., 2023). AMPylation also plays a crucial role in interactions between prokaryotes and eukaryotes. For instance, prokaryotes utilize this modification to influence host cell cytoskeletal and vesicular transport functions, thereby promoting their own proliferation (Yarbrough et al., 2009; Müller et al., 2010).

As research into AMPylation continues to advance, an increasing number of research teams are dedicating themselves to this field. Various methods for identifying AMPylation modifications have been developed, such as antibody detection, chemical labeling, and isotope labeling. However, the field still faces numerous challenges (Pieles et al., 2014; Höpfner et al., 2020; Kielkowski et al., 2020a). In-depth research on AMPylation modification helps reveal more intricate regulatory networks within cells, offering new perspectives on understanding the fundamental processes of life. It also provides potential new targets and approaches for the diagnosis, treatment, and drug development of related diseases.

## 2 AMPylation in prokaryotes

In the field of prokaryotes, early studies revealed that glutamine synthetase activity can be regulated by AMPylation, thereby influencing nitrogen metabolism (Shapiro et al., 1967; Brown et al., 1971). This discovery highlighted the crucial role of AMPylation in metabolic regulation within prokaryotes, laying the groundwork for subsequent in-depth research. Subsequent studies have demonstrated that AMPylation also participates in gene expression regulation in prokaryotes. For instance, bacteria release toxins that modify proteins involved in DNA replication through AMPylation, disrupting bacterial DNA topology and impairing bacterial growth (Harms et al., 2015). In addition to regulating their own gene expression, prokaryotes also suppress cell division by modifying non-self mitotic proteins through AMPylation, thereby mediating interspecies bacterial competition. Furthermore, prokaryotes maintain cellular redox homeostasis via AMPylation (Dudkiewicz et al., 2012; Wang et al., 2022). These findings significantly expand our understanding of AMPylation’s functions in prokaryotes. It has now been established that prokaryotes can regulate their own functions through AMPylation, primarily manifested in three aspects: controlling their own metabolism, modulating gene expression, and maintaining cellular redox homeostasis. **Table 1** summarizes three functional categories, which we will elaborate on below.

**Table 1:** The role of AMPylation in regulating self-associated functions in prokaryotes.

Name of the bacterium	ATPase	Substrate protein	Catalytic site	Downstream protein	Function
*E. coli*	GS-ATase	GS	Tyr397		Regulating nitrogen balance
*Corynebacterium glutamicum*		Glnk	Tyr51	AmtR	
*Bartonella*	Vbh TA	Gyrase Topoisomerase IV	GyrB:Tyr109 ParE:Tyr105		Inhibit bacterial DNA replication
*Fluorescent Pseudomonas*	Fic-1	Gyrase	Tyr109		
*Neisseria meningitidis*	NmFic	NmFic	Tyr183 Tyr188	Gyrase:Tyr109	Regulating bacterial growth
*Pseudotuberculosis Yersinia*	CccR	FtsZ	Thr8		

### 2.1 Regulation of self-metabolism

GS plays a crucial role in regulating nitrogen metabolism within organisms. *E. coli* reduces Gln synthesis under high-nitrogen conditions, while the opposite occurs under low-nitrogen conditions (Jiang et al., 1998). Research indicates that the metabolic conversion of PII regulatory proteins can mediate the AMPylation and de-AMPylation of GS, thereby regulating its enzymatic activity and intracellular nitrogen metabolism (Brown et al., 1971) (Jiang et al., 1998). Gs-ATase is the key enzyme catalyzing the AMPylation of GS. Gs-ATase contains a conserved G-X11-D-X-D motif, where the aspartic acid residue on this motif is the crucial conserved amino acid responsible for catalyzing the AMPylation modification of GS (Jiang et al., 2007). Gs-ATase contains two homologous adenyl transferase domains: AT-N and AT-C. The AT-C domain comprises a regulatory domain (R) and an adenyl transferase (AT) domain. Under high nitrogen conditions, increased Gln levels in *E. coli* activate Urydil-removing(UR) activity. The activated UR catalyzes the conversion of PII-UMP to PII. Subsequently, PII binds to the R domain, enhancing the enzymatic activity of the AT domain. This activity then catalyzes the AMPylation modification of GS, ultimately inhibiting its enzymatic activity and terminating glutamine synthesis. Conversely, in low-nitrogen conditions, decreased Gln levels in *E. coli* activate Urydil transferase(UTase) activity. UTase catalyzes the conversion of PII to PII-UMP. Upon binding PII-UMP to the R domain, the enzymatic activity of the AR domain increases, catalyzing the de-AMPylation of GS. This reactivates GS activity, initiating Gln synthesis (Brown et al., 1971).

GlnK is the homologue of the PII protein (Xu et al., 1998). In nitrogen metabolism regulation, AmtR functions as a transcription repressor that normally suppresses the expression of multiple genes involved in nitrogen metabolism. AMP-modified GlnK (adGlnK) acts as an inducer for AmtR, binding to AmtR to prevent its interaction with the DNA operator. This promotes the expression of related genes, thereby regulating bacterial nitrogen source utilization and metabolism. In *Bacillus glutamic acidus*, AmtR’s suppression of gene expression is only released when GlnK undergoes AMPylation at position 51 (Grau et al., 2021).

### 2.2 Regulation of gene expression

The toxin-antitoxin (TA) system in prokaryotes can interfere with protein translation to induce growth arrest, forming persister cells that tolerate stress (Page and Peti, 2016; Harms et al., 2018). The FicTA toxin-antitoxin module is a genetic element in bacteria composed of the FicT toxin and the FicA antitoxin. The FicT toxin disrupts the normal topological state of bacterial DNA through the enzymatic activity of its FIC domain, impairing normal physiological functions and inhibiting bacterial growth (Harms et al., 2017). The VbhTA complex from *Bartonella bacteria* belongs to the FicTA family, consisting of the VbhT toxin and VbhA antitoxin. The VbhTA module utilizes its Fic domain to induce AMPylation in DNA gyrase and topoisomerase IV. Specifically, the VbhT enzyme adds AMP molecules to the Tyr109 residue of the GyrB subunit of DNA gyrase and the Tyr105 residue of the ParE subunit of topoisomerase IV. Enzymes undergoing this AMPylation modification lose their inherent ATPase activity. This process disrupts critical steps in bacterial DNA supercoiling, unwinding, and knot-unraveling, thereby reversibly inhibiting bacterial growth (Harms et al., 2015). Overexpression of Fic protein leads to abnormal DNA topoisomerase activity, severely disrupting the DNA replication process. This triggers DNA damage and a replication stress response (Lu et al., 2020). Single-stranded DNA regions bind to RecA, activating its function. Activated RecA protein promotes the self-cleavage of LexA protein (Little, 1991). Furthermore, since LexA protein acts as an inhibitor of the SOS gene, its cleavage eliminates its inhibitory effect on the SOS gene, thereby activating the transcription of the SOS gene and triggering the SOS response (Sutton et al., 2000).

AMPylation can also trigger the SOS response by inhibiting DNA gyrase, aiding bacterial survival in adverse environments. In *Pseudomonas fluorescens*, Fic-1 interacts with the β subunit GyrB of DNA gyrase (Lu et al., 2020). Mass spectrometry and *in vivo* AMPylation experiments confirm that Fic -1 induces AMPylation at Tyr109 of GyrB. Mutations at this site or in Fic-1’s catalytic domain impair AMPylation activity, and Fic-1 primarily modifies the N-terminal domain of GyrB (Karimova et al., 1998) (Lu et al., 2016). Since DNA gyrase activity depends on ATP hydrolysis to introduce negative supercoils and relieve the tension generated by helicase during DNA replication, the reduced binding affinity of AMP-modified GyrB to ATP directly results in the loss of its ATPase activity. This subsequently prevents DNA gyrase from functioning normally, ultimately hindering bacterial DNA replication and leading to the exposure of single-stranded DNA and the formation of RecA filaments (Reece and Maxwell, 1991) (Little, 1991) (Nöllmann et al., 2007). Ultimately, promoting the self-cleavage of LexA protein and the subsequent onset of the SOS response (Sutton et al., 2000). It also discovered that GyrB undergoes auto-AMPylation at the Tyr5 site and identified AntF, an inhibitor of Fic-1. In bacteria, AntF suppresses Fic-1’s AMPylation activity on GyrB, thereby blocking Fic-1’s inhibition of DNA replication (Lu et al., 2016).

### 2.3 Regulating bacterial growth

AMPylation functions not only as a “molecular switch” regulating DNA gyrase but also as a self-activation switch for Fic protein, thereby releasing its own inhibition and regulating bacterial growth. The Class III Fic protein NmFic in *Neisseria meningitidis* undergoes self-AMPylation to release its own inhibition, thereby enabling AMPylation modification of GyrB. This process is termed cis-self-AMPylation of NmFic, representing a unique self-activation mechanism. Under normal conditions, the activity of wild-type NmFic (NmFic-wt) is inhibited due to the presence of an α-inhibitory domain (α-inh) that partially blocks the active site. For example, one strictly conserved Glu residue impedes ATP γ-P binding, thereby preventing effective substrate binding and maintaining NmFic in a self-inhibited state (Engel et al., 2012). Y183 is a key tyrosine residue in the α-inh of the NmFic protein, buried within the hydrophobic core region of the protein. When Y183 undergoes deacetylation, it partially unfolds the α-inh, thereby releasing its obstruction of the active site. This facilitates the transition of NmFic from its native deacetylated state to an acetylated form, enabling it to acetylate GyrB and consequently impact bacterial growth (Stanger et al., 2016). The CdFic modification in *Clostridium difficile* occurs independently of the inhibitory domain during the AMP-modification process (Dedic et al., 2016).

Additionally, the formation of NmFic tetramers obscures its binding site with the target protein, thereby hindering effective interaction between NmFic and the target protein. This subsequently inhibits its activity in performing AMP modification on the target protein. In contrast, experimentally engineered NmFic mutants, unable to form normal tetramers, exist as monomers and can perform AMP-modification on GyrB, affecting bacterial growth. A dynamic equilibrium exists between NmFic monomers and tetramers, influenced by factors such as ATP. In the presence of ATP, the transition of NmFic towards the tetrameric form is promoted. As NmFic concentration increases, the proportion of tetramers also rises, leading to more NmFic existing in the non-catalytically active tetrameric state and consequently reducing overall activity. This dynamic equilibrium mechanism allows NmFic activity to be finely tuned according to changes in the intracellular environment, adapting to the physiological needs of the cell (Stanger et al., 2016).

AMP modifications not only regulate the expression of their own genes but also confer competitive advantages by modifying key division proteins in non-host cells. CccR comprises an N-terminal FIC domain and a C-terminal DNA-binding domain. In *Yersinia pseudotuberculosis*, CccR functions as a dual-purpose T6SS toxin: it mediates intercellular communication as a transcription regulator in the kin cell, while also negatively regulating cccR gene expression by binding to its own promoter region via its DNA-binding domain. Simultaneously, it inhibits cell division in competing or neighboring cells by AMPylating the Thr residue of the cell division protein FtsZ, thereby mediating interspecies bacterial competition. FtsZ is a GTPase and tubulin homolog that self-assembles into a ring structure and generates contractile force. In most bacteria, FtsZ participates in cell division (Barrows and Goley, 2021). CccR interacts with FtsZ and induces its AMPylation at the Thr8 site, thereby inhibiting FtsZ’s GTPase activity and polymerization process. This disrupts the Z-ring structure, preventing cells from undergoing normal division. Consequently, cells continue to grow without dividing, ultimately forming a filamentous morphology (Wang et al., 2022).

### 2.4 Maintaining cellular redox homeostasis

SelO is a pseudokinase located in mitochondria that plays a role in oxidative stress responses (Dudkiewicz et al., 2012). SelO exhibits AMPylation activity, transferring the AMP molecule onto protein substrates rather than transferring the γ-phosphate group of ATP as classical kinases do. In classical kinases, the γ-phosphate group of ATP is the portion that binds to the protein substrate; whereas in SelO, the γ-phosphate group is flipped to the position where the α-phosphate group typically resides, and the α-phosphate group is flipped to the position where the γ-phosphate group typically resides (Bardwell, 2019). Both human SelO and yeast SelO are localized to mitochondria. When purified under non-reducing conditions, *E. coli* SelO exists as a dimer linked by an intramolecular disulfide bond, which modulates its AMP-modifying activity. Under reducing conditions or in the presence of thioredoxin, SelO adopts a monomeric state, at which point its AMP-modifying activity increases. SelO catalyzes the AMPylation modification of the E1 component sucA in the α-ketoglutarate dehydrogenase complex and glutaredoxin (grx) in *E. coli*, participating in cellular redox regulation by modulating the S-glutathionylation levels of proteins. Grx facilitates protein repair through deglutathionylation, mitigates oxidative stress damage to cells, and maintains cellular redox homeostasis, thereby playing a crucial role in cellular oxidative stress responses (Shelton et al., 2005) (Sreelatha et al., 2018).

## 3 AMPylation in eukaryotes

To date, over 600 modifications have been identified in mammalian cell proteins (http://www.uniprot.org/docs/ptmlist.txt). Beyond classical PTMs like phosphorylation, acetylation, ubiquitination, and methylation, AMPylation has gained increasing recognition as a crucial post-translational modification in eukaryotes. It triggers a series of pathophysiological processes and is implicated in diseases such as neurodegenerative disorders, cardiovascular diseases, and cancer. Furthermore, eukaryotes utilize AMPylation to regulate functions, including endoplasmic reticulum stress and immune defense (Mukherjee and Sreelatha, 2025).

### 3.1 Regulation of endoplasmic reticulum stress

**Figure 1:**
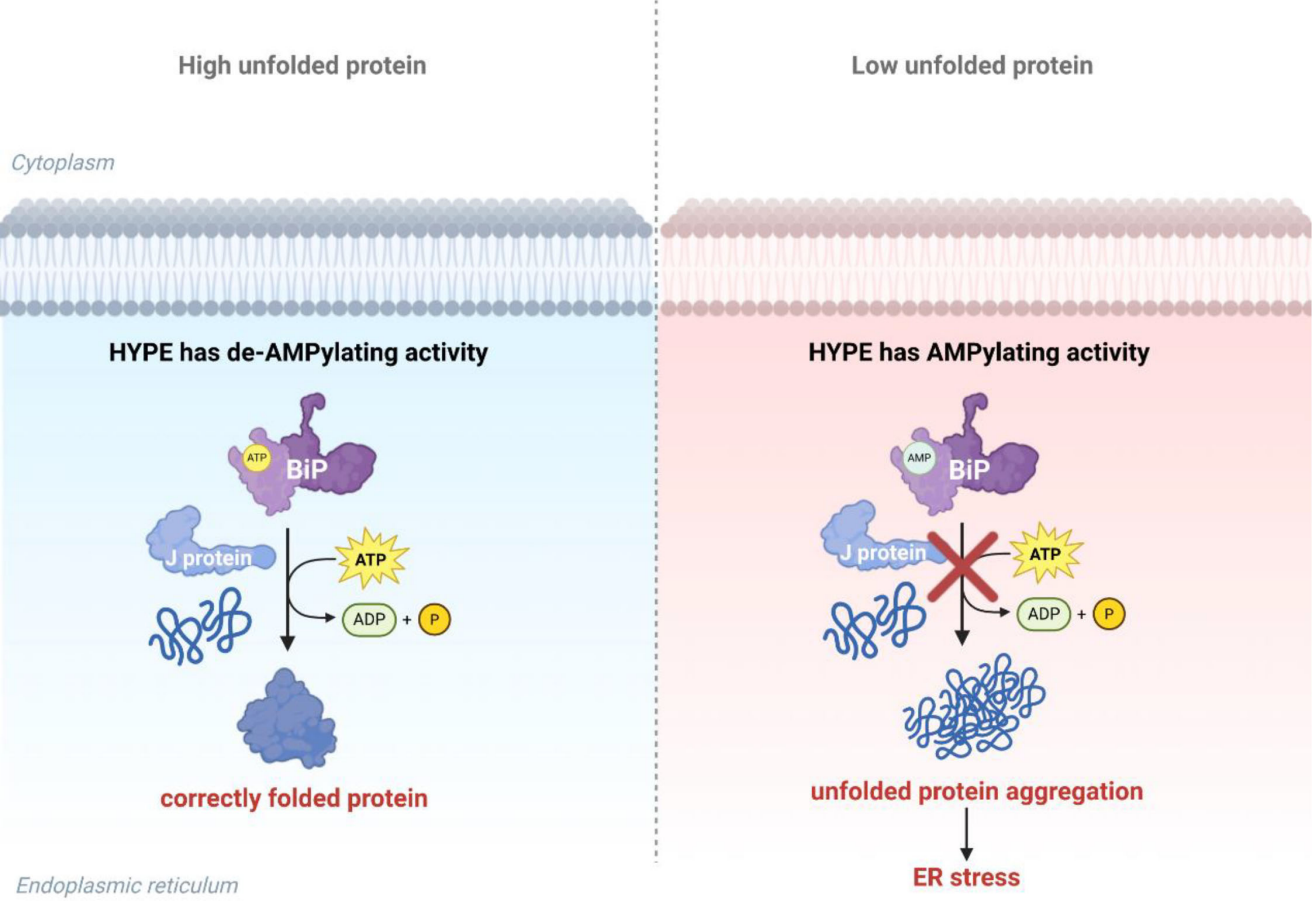
HYPE regulates ER unfolded protein fate. When unfolded proteins increase, HYPE exhibits de-AMPylating activity, allowing *BiP* to bind ATP and correctly fold proteins. When unfolded proteins decrease, HYPE exhibits AMPylating activity, leading to increased AMP-modified *BiP* that cannot function normally, thereby triggering endoplasmic reticulum stress.

HYPE (also known as FICD) is a 52 kDa protein containing a conserved Fic domain at its C-terminus (Worby et al., 2009). In humans and other eukaryotes, the HYPE protein functions as an enzyme with adenyl transferase activity, catalyzing the AMPylation of the endoplasmic reticulum molecular chaperone *BiP* at Ser365 and Thr366. This process alters *BiP* ‘s ATPase activity, playing a crucial role in maintaining endoplasmic reticulum homeostasis and regulating the unfolded protein response. As shown in **Figure 1**, J proteins, as auxiliary proteins for *BiP*, enhance folding efficiency by stimulating *BiP* to hydrolyze ATP (**Figure 1**) (Ham et al., 2014; Sanyal et al., 2015). The unfolded protein response is a cellular mechanism for coping with endoplasmic reticulum stress (ER stress), and thus the process of AMPylation significantly impacts the stress-response capacity of eukaryotic organisms (Wang et al., 2016).

AMPylation at the T366 site enhances *BiP*’s ATPase activity, while AMPylation at the T518 site inhibits its J-protein-assisted ATPase activity (Sanyal et al., 2021). AMPylation of *BiP* at Thr518 mediated by HYPE is an inactivation modification. AMPylation reduces *BiP*’s oligomerization capacity, makes it less susceptible to cleavage by SubA protease, and decreases its basal ATP hydrolysis rate by approximately 50%. Under normal conditions, the J-protein cofactor stimulates *BiP*’s ATP hydrolysis activity. However, AMPylated *BiP* is nearly completely resistant to J-protein-mediated ATP hydrolysis stimulation. When intracellular unfolded proteins are scarce, *BiP* undergoes AMPylation and inactivation, preventing hyperactive *BiP* from interfering with protein folding. When unfolded proteins increase, the AMPylation modification is removed, reactivating *BiP* to meet protein folding demands. Overexpression of active HYPE increases *BiP* AMPylation, thereby activating the UPR; conversely, HYPE knockout prevents *BiP* AMPylation, delaying UPR activation. This demonstrates that the AMPylation status of *BiP* modulates the activity of the UPR signaling pathway, influencing cellular responses to ER stress (Preissler et al., 2015).

The HYPE protein not only inactivates *BiP* through AMPylation modification at Thr518 but also reactivates it via de-AMPylation, meaning HYPE bidirectionally regulates *BiP*(**Figure 1**). AMPylation of *BiP* is regulated by Glu234 in the Fic domain, while de-AMPylation is triggered by proton transfer from His363 to the AMPylated Thr518 residue. This process plays a crucial role in maintaining protein folding homeostasis within the endoplasmic reticulum (Preissler et al., 2017a; Perera et al., 2021). FICD specifically binds to *BiP*’s NBD and conserved hydrophobic junction via its TPR motif, forming a complex. This specific binding preference enables FICD to interact preferentially with *BiP* in its ATP-bound state for AMPylation modification, thereby regulating *BiP* activity, rather than binding to *BiP* in other states (Fauser et al., 2021).

Similar to the mechanism by which HYPE catalyzes AMPylation of *BiP*, *Drosophila melanogaster*-derived dFic specifically modifies *BiP* with AMPylation when the number of unfolded proteins in the endoplasmic reticulum is low. This inactivates *BiP*, preventing its erroneous binding to and folding of unrelated proteins, thereby precisely maintaining endoplasmic reticulum protein folding. dFic catalyzes AMPylation of *BiP*’s Thr366 residue in a Ca^2+^-dependent manner under stress-free conditions *in vivo*. It preferentially catalyzes AMPylation of inactive *BiP*, which is rendered inactive by potentially interfering with the normal binding process between DnaJ family chaperone proteins and *BiP*, thereby preventing *BiP* from performing its chaperone function. Under stress conditions, when unfolded proteins increase, dFic de-AMPylates *BiP* to restore its normal function, ensuring proper protein folding within the endoplasmic reticulum (Ham et al., 2014). The site at which dFic protein catalyzes de-AMPylation is Thr518, but de-AMPylation does not depend on dFic protein dimerization; it only reduces the AMPylation activity toward the substrate *BiP* (Casey et al., 2018). dFic-mediated AMPylation of *BiP* interacts with endoplasmic reticulum stress to jointly maintain cellular homeostasis, thereby ensuring the normal function of the Drosophila visual system (Moehlman et al., 2018).

### 3.2 Participation in disease development

**Figure 2:**
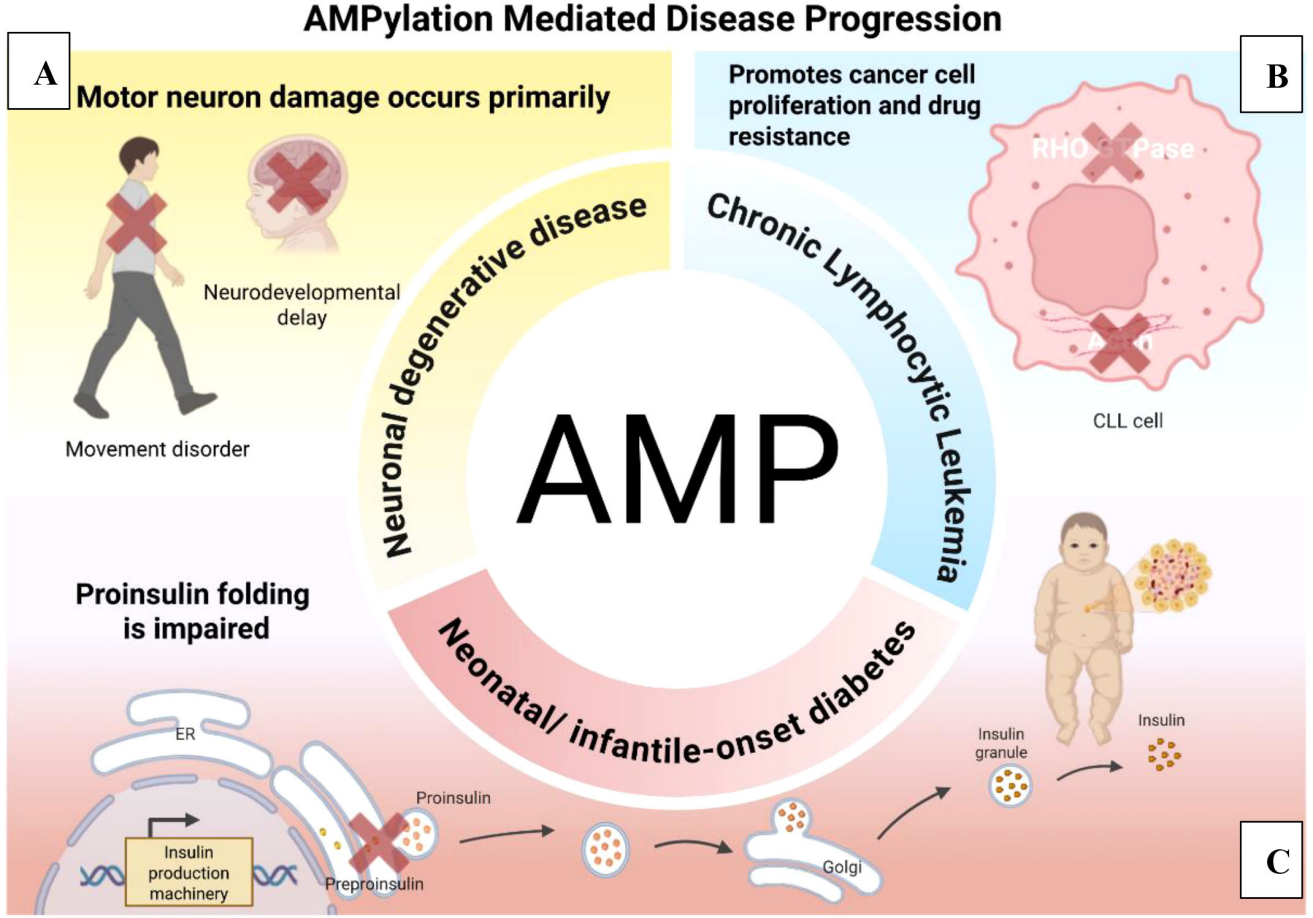
AMPylation mediated disease progression. **(A)** Mutations in the HYPE gene lead to accumulation of AMP-modified *BiP*, which inhibits *BiP* activity, thereby impairing protein folding, disrupting protein homeostasis, and ultimately contributing to neurodegenerative disease onset; HYPE modifies αSyn through AMPylation, thereby inhibiting its fibrillation, converting some fibrils from twisted to parallel conformations, and reducing membrane permeability to small unilamellar vesicles (SUVs), thus suppressing Parkinson’s disease progression. **(B)** In the pathogenesis of chronic lymphocytic leukemia, HYPE plays a crucial role by regulating proteins in the actin pathway and RHO GTPase pathway through AMPylation. **(C)** Mutations in the HYPE gene lead to abnormal intracellular levels of AMPylated *BiP*, impairing the body’s ability to effectively regulate *BiP* activity under stress conditions. This affects the insulin-secreting function of pancreatic β-cells, resulting in diabetes onset during the neonatal/infant period.

In recent years, research has revealed that abnormal AMPylation modifications are closely associated with the onset and progression of various diseases. First is neurodegenerative disease in motor neurons (**Figure 2**). The Arg374His mutation in HYPE disrupts its ATP-binding capacity, disrupting intramolecular interactions and leading to the accumulation of AMP-modified *BiP* protein—a phenomenon consistently validated in patient fibroblasts. This accumulation is accompanied by weakened FICD de-AMPification activity and increased sensitivity to endoplasmic reticulum stress. This abnormal accumulation of AMP-modified *BiP* inhibits its molecular chaperone function, thereby impairing protein folding, disrupting protein homeostasis, and ultimately causing neurodegeneration in motor neurons (Rebelo et al., 2022). Additionally, the Arg371Ser mutation in HYPE causes a novel genetic syndrome with a similar pathogenesis. This mutation impairs the timely AMPylation and de-AMPylation of *BiP*, leading to abnormal intracellular levels of AMPylated *BiP*. Under stress conditions, *BiP* activity cannot be effectively regulated, impairing the function of proteins involved in normal endoplasmic reticulum folding within insulin-producing β-cells and neurodevelopment-related cells. This results in neonatal/infantile-onset diabetes (**Figure 2**) (Perera et al., 2023). In addition, in chronic lymphocytic leukemia, HYPE influences cell proliferation and carcinogenic processes by regulating proteins in the actin pathway and RHO GTPase pathway through AMPylation (**Figure 2**) (Fatima et al., 2024).

Beyond inducing disease, AMPylation can also suppress disease progression. HYPE modifies αSyn through AMPylation, primarily at residues T33, T54, and T75 on its N-terminus. AMPylation of αSyn inhibits its fibrillation, converts some fibrils from twisted to parallel conformations, and reduces membrane permeability to small unilamellar vesicles (SUVs). Neurons employ these adaptations to counteract αSyn toxicity and shield themselves from potential stress-induced damage. This process inhibits Parkinson’s disease (PD) progression, offering novel therapeutic avenues for PD treatment (Sanyal et al., 2019).

AMPylation in eukaryotes also influences neuronal differentiation (**Figure 2**). For example, during neuronal differentiation and maturation, AMPylation acts as a novel post-translational modification of lysosomal proteins, specifically regulating lysosomal proteins such as PLD3 and ACP2. Notably, soluble PLD3 exhibits significantly elevated AMPylation levels during neuronal maturation. This modification inhibits PLD3’s 5’-exopeptidase activity, thereby regulating lysosomal function and neural development, and further contributing to the progression of neurodegenerative diseases (Becker et al., 2021). The distinct AMPylation levels observed in transport proteins and cytoskeletal proteins within neurons compared to other cellular contexts suggest that AMPylation may participate in cell type specialization and the differentiation process from human-induced pluripotent stem cells (iPSCs) through neural progenitor cells (NPCs) to neurons. Furthermore, under conditions of endoplasmic reticulum stress, AMPylation responses differ across distinct cell types (Kielkowski et al., 2020b).

### 3.3 Participation in immune regulation

#### 3.3.1 Regulation of innate immunity

In *Caenorhabditis elegans*, the enzyme Fic-1 catalyzes the AMPylation of HSP-1 and HSP-3 from the Hsp70 family, the translation elongation factor eEF-1A2, and histones H2 and H3. This process influences the nematode’s innate immunity against *Pseudomonas aeruginosa* and endoplasmic reticulum signaling functions (Harding et al., 2000). The nematode FIC-1 modifies HSP-1 with AMPylation, preventing its normal folding of DAF-7 and DBL-1. This inhibits signal molecule production, thereby disrupting TGF-β signaling in *Caenorhabditis elegans* and affecting nematode development, behavior, and immune regulation (Hernandez-Lima et al., 2022). In innate immune responses, AMP modification may indirectly enhance nematode immune defenses by promoting the dissociation of chaperone proteins from their intrinsic binding partners, thereby activating the unfolded protein response, as well as by regulating protein translation processes (Truttmann et al., 2016).

#### 3.3.2 Regulation of adaptive immunity

Mouse mFICD exerts a significant influence on B cell development and function within the immune system. By modifying *BiP* through AMPylation, it impacts immunoglobulin folding and secretion, thereby ensuring normal antibody production (Hendershot et al., 1987). Furthermore, mFICD deficiency causes most AMP-modified proteins in mice to lose their AMP modification. This process alters protein secretion in splenic cells, such as reducing LPS-induced IL-6 secretion and increasing IL-1β production, thereby regulating the mouse immune system. In the nervous system, researchers observed an enhanced tendency in mFICD-deficient mice for visual non-spatial short-term learning. This indicates that mFICD deficiency impacts neuronal plasticity. Although the precise mechanism remains unclear, it may be associated with sustained disinhibition of *BiP* and the role of protein homeostasis regulation within neurons (McCaul et al., 2021).

### 3.4 Similarities and differences with prokaryotic AMPylation

AMPylation achieves precise regulation of biological processes by modifying the active sites of target proteins or altering their subcellular localization. This core mechanism is highly conserved across both prokaryotic and eukaryotic organisms. Whether it is the inhibition of GS catalytic activity through AMPylation at Tyr397 in prokaryotes or the inactivation of *BiP* via AMPylation at Thr518 in eukaryotes, regulation occurs by directly acting upon functionally critical sites of the target protein (Jiang et al., 2007; Preissler et al., 2015). Additionally, “reversibility” is another common feature of AMPylation across species. In prokaryotes, AMPylation and de-AMPylation of GS are mediated by distinct domains of GS-ATase, while in eukaryotes, HYPE catalyzes both modification and demodification of *BiP*. This reversibility enables cells to rapidly switch the functional state of target proteins in response to environmental changes—such as nitrogen concentration fluctuations in prokaryotes or endoplasmic reticulum stress levels in eukaryotes—thereby maintaining systemic homeostasis (Jiang et al., 1998; Preissler et al., 2017a).

Unlike AMPylation in prokaryotes, which primarily focuses on self-metabolic balance, gene expression regulation, and interspecies competition, this modification in eukaryotes functions more toward maintaining the complex homeostasis of multicellular organisms and regulating disease progression. Prokaryotic AMPylation often regulates single metabolic pathways through simple “enzyme-substrate” linear interactions, such as GS-ATase regulating nitrogen metabolism (Jiang et al., 2007). In eukaryotes, this modification often forms multidimensional regulatory networks—it can directly modify molecular chaperones like *BiP* to regulate endoplasmic reticulum stress, and also participate in neuronal differentiation and neurodegenerative diseases by modifying α-synuclein, lysosomal proteins, and others (Ham et al., 2014; Sanyal et al., 2019; Becker et al., 2021). From the perspective of regulatory targets, the substrates of AMPylation in prokaryotes are predominantly core functional proteins such as the metabolic enzyme GS and the DNA replication-associated protein GyrB (Jiang et al., 2007; Harms et al., 2015). The substrate coverage of eukaryotes is broader, encompassing molecular chaperones such as *BiP*, cytoskeletal-associated proteins, immune signaling molecules, and others (Sanyal et al., 2015; Kielkowski et al., 2020b; Hernandez-Lima et al., 2022). AMPylation in eukaryotes often synergizes with other post-translational modifications such as ubiquitination to achieve more precise functional regulation (Woolery et al., 2014).

## 4 Prokaryotes regulate eukaryotes through AMPylation

In the interactions between prokaryotes and eukaryotes, AMPylation has emerged as a unique modification mechanism attracting increasing attention from researchers. Prokaryotes precisely regulate eukaryotic biological processes through AMPylation. For instance, certain bacteria secrete specific enzymes that attach AMP molecules to lysine or serine residues on eukaryotic proteins. This process significantly impacts key proteins within eukaryotic cells—such as cytoskeletal proteins and vesicle transport-associated proteins—thereby affecting host cell function.

### 4.1 Bacterial AMP-modified molecules inhibit host cell cytoskeletal function

The cytoskeleton plays a crucial role in vital activities such as maintaining cell morphology, cell motility, and cell division. Bacteria disrupt the cytoskeletal structure by AMP-modifying host cell proteins, thereby activating or inhibiting signaling pathways and suppressing host-related biological functions.

**Figure 3:**
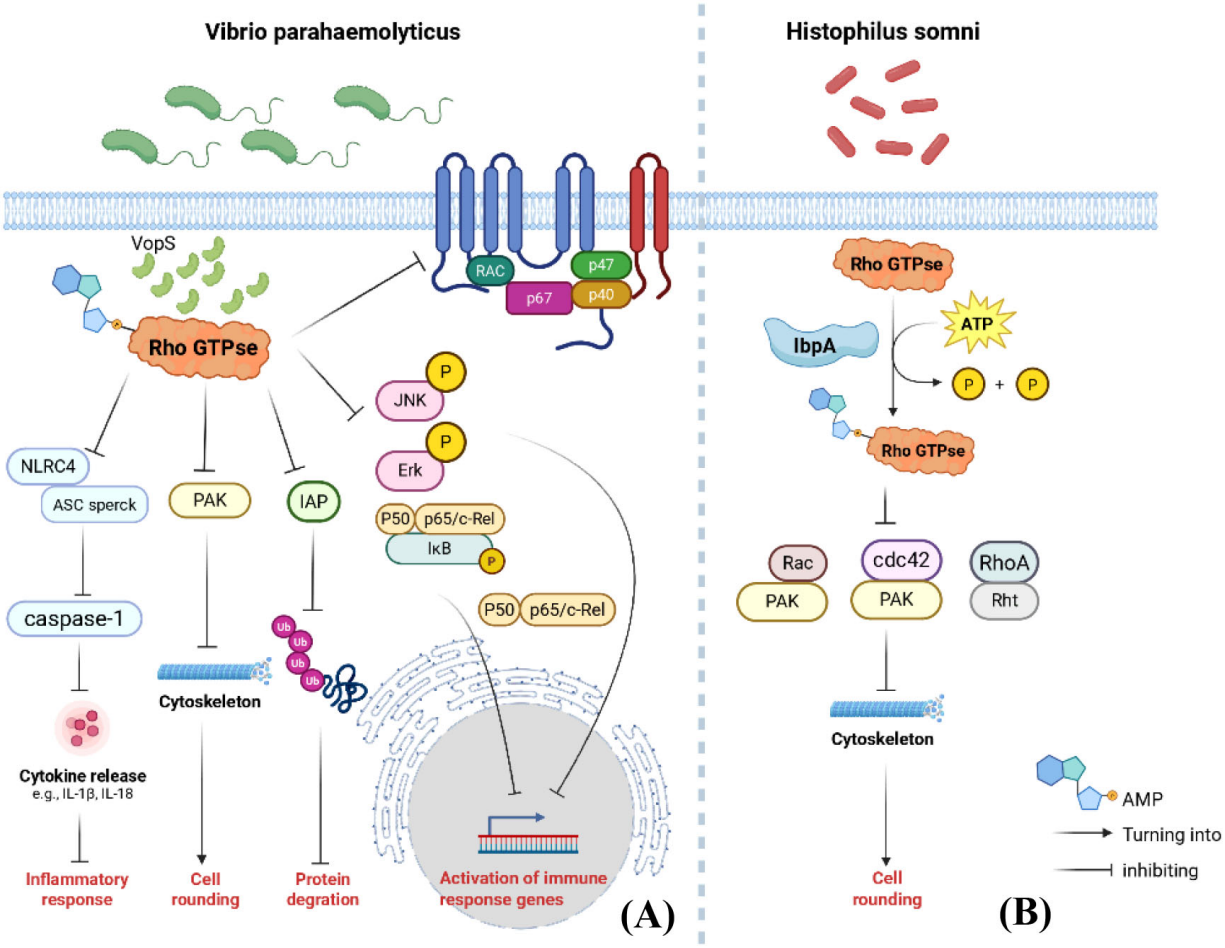
Mechanism diagram of prokaryotic AMPylation regulating eukaryotic-related functions. **(A)** VopS inactivates Rho GTPases through AMPylation, disrupting ASC speck assembly. This prevents effective recruitment and activation of the NLRC4 inflammasome to caspase-1, reducing proinflammatory factor release and weakening the host’s inflammatory response to bacterial infection. VopS AMPylation disrupts the binding between Rho GTPases and PAK, preventing PAK activation. resulting in uncontrolled actin rearrangement and loss of normal cell morphology; VopS directly blocks the interaction between Rho GTPases and IAP through AMPylation, preventing IAP from mediating GTPase ubiquitination via E3 ubiquitin ligase activity and reducing degradation; VopS blocks activation of NF-κB, JNK, and Erk pathways by AMP-modifying Rho GTPases, thereby inhibiting transcription of target genes including pro-inflammatory and anti-apoptotic factors; VopS disrupts Nox2 binding to Rho GTPases through AMP-modification, suppressing superoxide production. **(B)** The IbpA protein modifies host proteins via AMPylation, blocking Rac-PAK, RhoA-Rht, and Cdc42-PAK interactions. This inhibits actin cytoskeletal assembly in host cells, leading to cell rounding and death.

The C-terminal region of *Vibrio parahaemolyticus* VopS protein contains an Fic domain that catalyzes AMPylation modification of the conserved Thr35 in the Switch I region of Rho GTPase family proteins Rac1, RhoA, and Cdc42 (**Figure 3**) (Yarbrough et al., 2009). This inactivates them, thereby disrupting NF-κB, Erk, and JNK signaling pathways. It prevents effector proteins from binding to IAP proteins, inhibits E3 ubiquitin ligase-mediated degradation of effector proteins, and suppresses superoxide production by the NOX2 complex (**Figure 3**) (Woolery et al., 2014). Consequently, it modulates cellular immune activation of the pyrin inflammasome while simultaneously inhibiting NLRC4-mediated inflammasome activation, ultimately leading to cytoskeletal collapse and cell death (**Figure 3**) (Xu et al., 2014; Higa et al., 2013). Another study revealed that VopQ is the first effector protein to act, interacting with the host cell’s V-ATPase to cause lysosomal deacidification and inhibit fusion between lysosomes and autophagosomes. This activates the host cell’s unfolded protein response, specifically the Ire1 signaling pathway, which in turn activates the ERK1/2 signaling pathway and promotes host cell survival signals. Subsequently, the VopS effector protein intervenes by inhibiting Rho GTPase activity through AMPylation, thereby suppressing the VopQ-induced ERK1/2 signaling pathway and causing host cell death. This activation and inhibition of signaling pathways mediated by VopQ and VopS demonstrates how pathogens finely regulate host cell signaling to advance their infection process (De Nisco et al., 2021). VopS-mediated AMP modification exerts multifaceted effects on the function of novel substrates, primarily by disrupting their interactions with effector proteins, altering intracellular localization, and influencing physiological processes such as cytoskeletal regulation, cell migration, invasion, and endocytosis. These changes enable bacteria to manipulate host cell physiology during infection, thereby promoting their own survival and spreading the infection (Rauh et al., 2020).

The C-terminal region of the IbpA protein from *Histophilus somni* contains two Fic domains plus a YopT-homologous domain, with YopT harboring three conserved catalytic amino acid residues: Cys, His, and Asp. Upon entering host cells, the Fic domains catalyze AMPylation modifications at Tyr32 of host Rac protein, Tyr34 of RhoA protein, and Tyr32 of Cdc42 protein. These modifications sequentially block the interactions between Rac and PAK, RhoA and Rht, and Cdc42 and PAK, thereby inhibiting actin assembly in host cells and ultimately causing cell rounding and death (**Figure 3**) (Xiao et al., 2010; Worby et al., 2009). Additionally, the secretory protein PfhB2 from the opportunistic pathogen *Pasteurella multocida* shares structural similarity with IbpA and also possesses AMPase activity targeting Rho GTPases (Mattoo et al., 2011).

**Figure 4:**
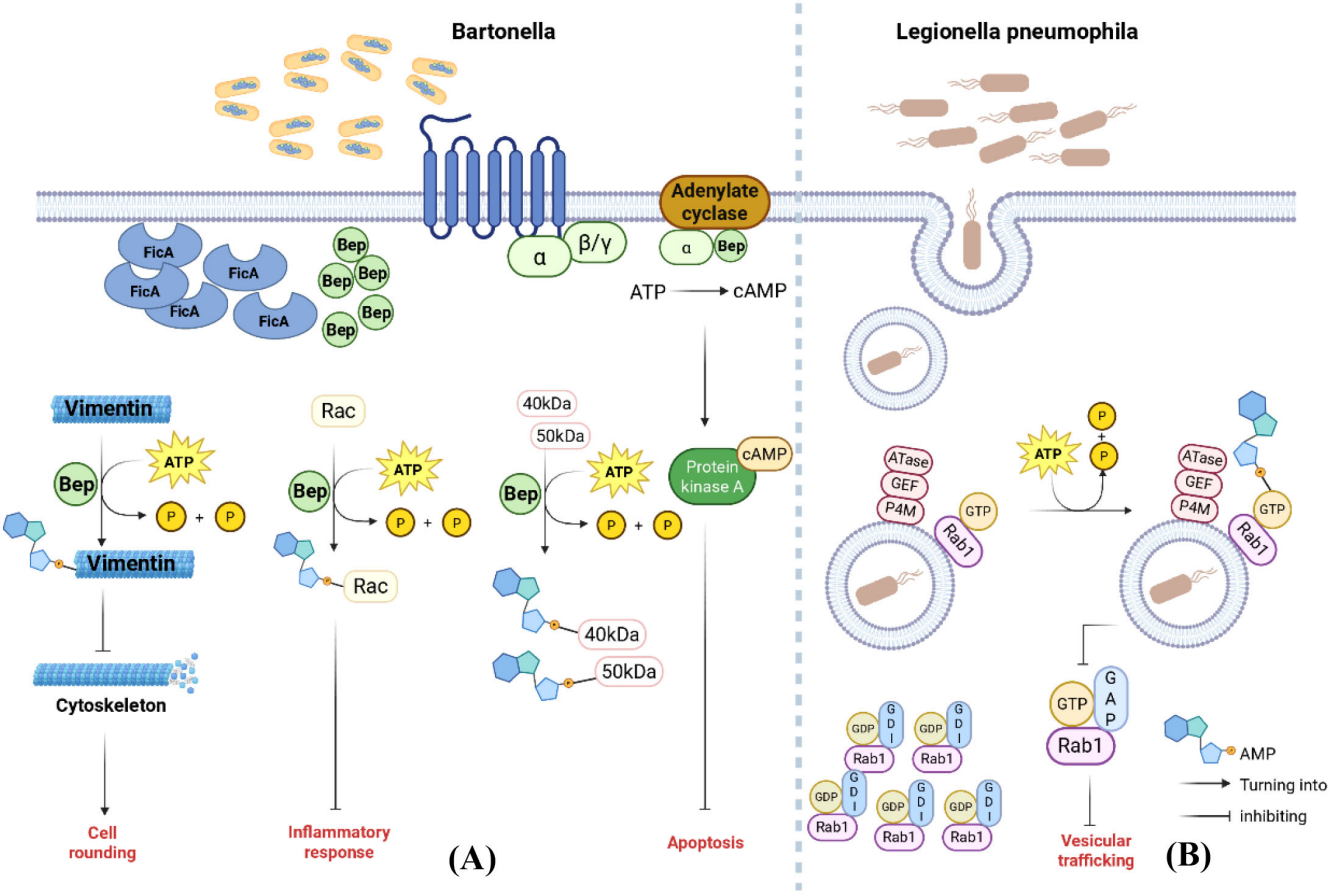
Mechanism diagram of prokaryotic AMPylation regulating eukaryotic-related functions. **(A)** Beps and the FicA antitoxin form a stable complex in *Bartonella* species. After Bep is transported into the host cell, the FicA antitoxin dissociates from Bep, allowing the Bep protein to exert its effects; Bep2 can AMPylate the host cell’s vimentin protein, altering the host cell structure. BepA not only binds to Gαs to elevate intracellular cAMP levels but also AMPylates unknown 40- and 50-kDa target proteins, thereby inhibiting endothelial cell apoptosis to establish chronic infection; Bep1 AMPylates GTPases of the Rac subfamily to evade the host innate immune response. **(B)***Legionella pneumophila*’s DrrA protein modifies Rab1-GTP via AMPylation to block its binding to GAP, inhibiting deactivation and ultimately maintaining Rab1’s sustained activation. This disrupts vesicular transport between the host cell’s endoplasmic reticulum and Golgi apparatus.

Additionally, in *Bartonella rochalimae*, the secreted protein Bep2 is capable of AMPylating the host cell’s vimentin protein. This AMPylation modification suggests that Bep2 may promote pathogen infection and survival by altering the structure and function of host cells (**Figure 4**) (Pieles et al., 2014).

This regulatory strategy enables bacteria to establish a survival advantage within host cells, thereby facilitating their proliferation.

### 4.2 Regulation of chronic infection by bacterial amide modification

Pathogens modify host proteins or themselves through AMPylation via the Fic domain of effector proteins, thereby disrupting host cellular processes such as apoptosis, immune responses, and gene expression. Simultaneously, they precisely regulate the spatiotemporal specificity of these modifications through mechanisms like antitoxins, facilitating their own colonization and the establishment of chronic infections.

*Bartonella henselae* can invade human endothelial cells through two distinct mechanisms: one involves passive phagocytosis to form bacterial-containing vesicles (BCVs), while the other involves active invasion of internalized bacteria. The effector protein Bep participates in the AMPylation modification of host proteins via its N-terminal Fic domain (Palanivelu et al., 2011). The BepA protein produced by *Bartonella henselae* not only binds to Gαs and adenylate cyclase (AC) to elevate intracellular cAMP levels, but also efficiently attaches AMP to 40-kDa and 50-kDa proteins, thereby inhibiting endothelial cell apoptosis to establish chronic infection (**Figure 4**) (Pulliainen et al., 2012; Schmid et al., 2006).

Bep forms a stable complex with the FicA antitoxin, which inhibits BepFIC’s AMPylation activity and participates in its transport through interaction with BepFIC. After BepFIC is transported into the host cell, the FicA antitoxin dissociates from BepFIC, thereby enabling the Bep protein to exert its activity within the eukaryotic target cell (**Figure 4**). This mechanism ensures the Bep protein precisely reaches its target site and exerts its regulatory effects on the host cell at the appropriate time and under the appropriate conditions, reflecting *Bartonella*’s fine-tuned regulation of the host cell infection process.

Additionally, Bep1 secreted by *Bartonella* exhibits high selectivity among numerous Rho family small GTPases, specifically targeting GTPases of the Rac subfamily for AMPylation modification, such as Rac1, Rac2, Rac3, and RhoG (**Figure 4**). While not acting on closely related Cdc42 or RhoA subfamilies. Bep1’s targeting selectivity for Rac subfamily GTPases is achieved through interactions between Bep1 and N-terminal residues of the Rho insertion helix, as well as residues within the G4 motif. This process enables *Bartonella* to evade the host’s innate immune response by avoiding activation of immune-associated inflammasomes, thereby establishing chronic asymptomatic infections (Dietz et al., 2021).

*Coxiella burnetii* evades host responses by secreting effector proteins. In the absence of DNA, CbFic2 exists as a monomer and exhibits AMPylase activity, modifying histones through AMPylation. This modification may participate in gene expression regulation and chromosomal structural maintenance. In the presence of DNA, CbFic2 dimerizes and exhibits deAMPylase activity. Additionally, CbFic2 can self-modify in the absence of DNA, but this modification is inhibited upon DNA binding (Höpfner et al., 2023). This dynamic regulation helps bacteria evade host immune surveillance and establish persistent infections.

### 4.3 Bacteria influence host vesicle transport through AMPylation

Vesicular transport serves as a crucial pathway for intracellular material transport and signal transduction. Bacteria interfere with vesicular transport by modifying host vesicle transport-related proteins through AMPylation, thereby disrupting material metabolism and signal transduction within host cells. The specific mechanism is shown in **Figures 3**, **4**.

The occurrence of AMPylation modification catalyzed by *Legionella pneumophila* DrrA protein requires the cooperative action of three distinct domains: the N-terminal adenosyltransferase (ATase) domain, the central guanine nucleotide exchange factor (GEF) domain, and the C-terminal P4M domain (Müller et al., 2010; Murata et al., 2006; Brombacher et al., 2009). DrrA binds to LCV membranes via its P4M domain. In the cytoplasm, GDP-dissociating inhibitor (GDI) binds to GDP-bound Rab proteins, thereby inhibiting Rab1 activity. DrrA’s GEF recruits Rab1 to the LCV membrane and catalyzes its conversion to Rab1-GTP. DrrA’s Atase modifies Rab1-GTP through AMPylation, blocking its interaction with downstream GTPase-activating proteins (GAPs). This inhibits Rab1 deactivation, leading to sustained Rab activation and disrupting vesicular transport between the endoplasmic reticulum and Golgi apparatus in host cells. As shown in **Figure 4**, *Legionella pneumophila* inhibits Rab1 inactivation and maintains its sustained activation state by AMP-modifying Rab1-GTP. This is consistent with the previously described mechanism whereby AMP modification affects vesicle transport (**Figure 4**) (Müller et al., 2010).

Within the AMPylation domain of DrrA, there exists a non-canonical Rab-binding site (NC-RBS) located opposite the catalytic center. Rab1 can remotely activate DrrA’s AMPylation activity by binding to this site. When unbound to Rab1, the catalytic site of DrrA remains in a disordered, inactive state, unable to effectively bind ATP and catalyze AMP transfer; Only when Rab1-GTP binds to the NC-RBS does an allosteric effect drive the reconfiguration of the DrrA catalytic site into an ordered, active state. This simultaneously promotes the proximity of Rab1 to the DrrA catalytic site, ensuring modification occurs exclusively on Rab1 rather than randomly affecting other host proteins (Mondino et al., 2020; Du et al., 2021). Additional research has revealed that *Legionella pneumophila* secretes a membrane translocation protein, SidD, encoded by an open reading frame adjacent to the sidM gene, comprising 507 amino acids (Chen et al., 2013). SidD catalyzes the removal of the AMP moiety from Rab1-GTP through de-AMPylation. Subsequently, Rab1-GTP binds to the downstream factor LepB, converting Rab1-GTP into the inactive form Rab1-GDP. This inactivated form is then released from the membrane to participate in vesicle transport (Tan and Luo, 2011; Neunuebel et al., 2011).

Additionally, SidJ is an effector protein secreted by *Legionella pneumophila*. Through dual modifications of AMPylation and glutamylation, it precisely regulates the activity of SdeA, a core member of the homologous effector protein family SidE (Kotewicz et al., 2017; Bhogaraju et al., 2016). The AMPylation process catalyzed by SidJ requires the participation of the eukaryotic-specific protein calmodulin (CaM). In reactions where CaM is present, SidJ undergoes self-AMPylation. When using ³²P-α-ATP in experiments, a strong signal of SidJ self-AMPylation is detectable in reaction systems containing CaM. This self-AMPylation phenomenon becomes more pronounced in the absence of glutamate or modifiable SdeA. This indicates that SidJ favors self-AMPylation when no suitable substrate is available for glutamylation. Conversely, when SdeA is present with glutamate, SdeA undergoes AMPylation modification, a process that interferes with SidJ’s self-AMPylation. Experimental results show that in reactions involving SdeA, glutamate, and SidJ simultaneously, the degree of SidJ’s auto-AMPylation is significantly reduced, indicating a competitive relationship between SdeA’s AMPylation modification and SidJ’s auto-AMPylation. CaM first binds to SidJ, activating its enzymatic activity, which then leads to the AMPylation of SdeA’s Glu860 residue, forming an unstable E860-AMP intermediate. Subsequently, the amino group of free glutamate undergoes a nucleophilic attack on the activated carbonyl group of this intermediate, ultimately leading to glutamylation at the Glu860 site and the release of AMP. This mechanism inhibits SdeA-related activity, preventing the adverse effects of excessive SidE activation on bacterial infection and allowing the restoration and maintenance of normal cellular physiological functions to a certain extent (Gan et al., 2019).

## 5 The application of AMPylation

AMPylation, as a dynamic post-translational modification of proteins, has demonstrated broad application potential in recent years across research fields such as disease treatment and cell biology. In the identification of AMPylation, highly sensitive and specific monoclonal antibodies enable the specific detection of AMPylated proteins, providing a reliable tool for functional studies regardless of protein background (Höpfner et al., 2020). Fluorescence polarization-based high-throughput screening methods can detect changes in HYPE enzyme AMPylation activity, facilitating the identification of enzyme activators and inhibitors (Camara et al., 2020). Additionally, cell-permeable nucleotide probes combined with click chemistry enable dynamic imaging and chemical proteomics analysis of AMP-modified proteins in live cells, laying the foundation for *in vivo* functional studies (Kielkowski et al., 2020a).

In constructing disease models, the progression of disease onset and development can be simulated by modulating the activity of relevant AMP-activating proteases or altering the AMPylation status of proteins. For instance, regulating FICD activity in neurons to alter protein AMPylation levels helps investigate its roles in neurogenesis, neuronal differentiation, and the function of disease-associated proteins (Kielkowski et al., 2020b). Studies on FicD’s deAMPylation function and its impact on endoplasmic reticulum homeostasis also enable the construction of models for diseases such as pancreatic endocrine dysfunction and diabetes (Casey et al., 2025). These disease models provide powerful tools for exploring the underlying mechanisms of related pathological processes.

In the field of protein function research, AMPylation can alter functional properties of proteins such as stability, enzyme activity, and cofactor binding. Inducing or inhibiting AMPylation to observe changes in protein function helps deepen understanding of the roles of target proteins in biological processes. For example, studies have confirmed that AMPylation regulates the functions of glutamine synthetase and Rab proteins, providing insights into the molecular mechanisms of cellular metabolism and signal transduction (Anderson and Stadtman, 1970; Du et al., 2021).

In the field of disease treatment and drug development, targeting AMPylation-related enzymes or proteins based on their pathogenic mechanisms can achieve therapeutic goals. For example, small-molecule inhibitors targeting abnormal AMPylation can restore *BiP* function, providing a potential therapeutic strategy for diseases such as neonatal diabetes (Chatterjee et al., 2025).

## 6 Discussion

As biochemical research continues to advance, AMPylation has gained increasing attention as an emerging form of post-translational protein modification. Due to its crucial roles in cellular metabolic regulation and influencing cytoskeletal structures, research in this field has become one of the current hotspots in the scientific community. However, compared to traditional studies on post-translational modifications, comprehensively elucidating the mechanisms and functions of AMPylation still faces numerous challenges.

First, the regulatory mechanisms of AMPylation remain incompletely understood, involving a diverse array of modified proteins with uncertain modification sites. For example, the AMPylation activity of Drosophila Fic is regulated by its own dimerization state, whereas its de-AMPylation activity can be exerted without dimerization. The dynamic regulatory signals governing this dimerization state remain unidentified (Casey et al., 2018) Second, the relationship between AMPylation structure and function is complex, as different modification patterns and sites may yield distinctly different biological effects. The precise mechanism by which upstream activation signals of AMP-activating enzymes, such as ER stress, switch the AMPylation/de-AMPylation activities of HYPE remains unclear (Preissler et al., 2015; Perera et al., 2021). Third, current AMPylation research predominantly focuses on static aspects, whereas the process in living organisms is inherently dynamic. Dynamic AMPylation refers to the reversible regulatory process of AMPylation modification under physiological or pathological conditions, mediated by modifying enzymes and de-modifying enzymes to maintain the balance of intracellular signaling networks. HYPE in eukaryotes and GS-ATase in prokaryotes have been demonstrated to bidirectionally regulate *BiP* through both AMPylation and deAMPylation. However, this dual-functionality remains poorly characterized in other AMPylases such as SidJ (Gan et al., 2019). Future studies may further elucidate whether these enzymes also exhibit similar dual-functionality. Developing dynamic research methodologies represents an inevitable future direction.

Compared to another review focusing on the molecular mechanisms of AMPylation core enzymes and their association with classic diseases, this review achieves multidimensional expansion and deepening in content (Mukherjee and Sreelatha, 2025). It introduces new mechanisms of AMPylation regulation in prokaryotes, such as its role in bacterial growth and interspecies competition, thereby refining the prokaryotic functional network. It extends the functional boundaries of eukaryotic AMPylation, adding examples like lysosomal proteins in neuronal differentiation. It refines prokaryote-eukaryote interaction mechanisms, revealing how bacteria establish chronic infections through AMPylation and synergistically regulate host signaling pathways—such as the relay action of VopQ-VopS. Furthermore, this review introduces new application directions, covering disease model construction, targeted drug development, and novel detection technologies, thereby bridging basic research and clinical translation. This review not only breaks away from the traditional linear “enzyme-substrate-disease” framework but also focuses on “regulatory networks + cross-species interactions + applied value,” providing a more comprehensive perspective for functional exploration and translational research in the field of AMPylation (Mukherjee and Sreelatha, 2025). Despite these advancements, unresolved issues persist: for example, the specific regulatory network of AMPylation in neurodegenerative diseases remains unclear, and current studies mostly focus on individual target proteins rather than analyzing the overall regulatory network (Sanyal et al., 2019). Although it is known that prokaryotes can regulate host cells through AMPylation—such as *Legionella*’s DrrA modifying Rab1—whether host eukaryotic cells possess “anti-AMPylation” defense mechanisms and how AMPylation signals interact across species remain unexplored research areas (Müller et al., 2010). Existing models also have limitations, such as the lack of *in vivo* dynamic tracking tools for AMPylation. Currently identified AMPylated substrates like *BiP* and grx represent only the tip of the iceberg, with numerous low-abundance substrates remaining undiscovered due to detection technology constraints (Höpfner et al., 2020). Emerging directions include the development of highly specific AMPylation detection tools and the exploration of AMPylation-targeted drugs for rare diseases.

Nevertheless, with the rapid advancement of biochemistry, molecular biology, and related fields, scientists are poised to unravel the mechanisms of AMPylation more profoundly and uncover additional biological functions associated with it in the near future. This will enhance our understanding of AMPylation’s role in life processes, offering novel approaches and methods for disease diagnosis, treatment, and prevention, ultimately benefiting humanity.
